# Photoreceptor metabolic reprogramming: current understanding and therapeutic implications

**DOI:** 10.1038/s42003-021-01765-3

**Published:** 2021-02-24

**Authors:** Warren W. Pan, Thomas J. Wubben, Cagri G. Besirli

**Affiliations:** Department of Ophthalmology and Visual Sciences, Kellogg Eye Center, University of Michigan, Ann Arbor, MI USA

**Keywords:** Translational research, Mechanisms of disease, Retina

## Abstract

Acquired and inherited retinal disorders are responsible for vision loss in an increasing proportion of individuals worldwide. Photoreceptor (PR) death is central to the vision loss individuals experience in these various retinal diseases. Unfortunately, there is a lack of treatment options to prevent PR loss, so an urgent unmet need exists for therapies that improve PR survival and ultimately, vision. The retina is one of the most energy demanding tissues in the body, and this is driven in large part by the metabolic needs of PRs. Recent studies suggest that disruption of nutrient availability and regulation of cell metabolism may be a unifying mechanism in PR death. Understanding retinal cell metabolism and how it is altered in disease has been identified as a priority area of research. The focus of this review is on the recent advances in the understanding of PR metabolism and how it is critical to reduction-oxidation (redox) balance, the outer retinal metabolic ecosystem, and retinal disease. The importance of these metabolic processes is just beginning to be realized and unraveling the metabolic and redox pathways integral to PR health may identify novel targets for neuroprotective strategies that prevent blindness in the heterogenous group of retinal disorders.

## Abstract (web summary)

Photoreceptor death is central to vision loss in various retinal diseases. Disruption of nutrient availability and cell metabolism may underlie photoreceptor death. In this review, Pan et al. focus on the recent advances in the understanding of photoreceptor metabolism and suggest novel targets for neuroprotective strategies that prevent blindness.

## Introduction

**Fig. 1:**
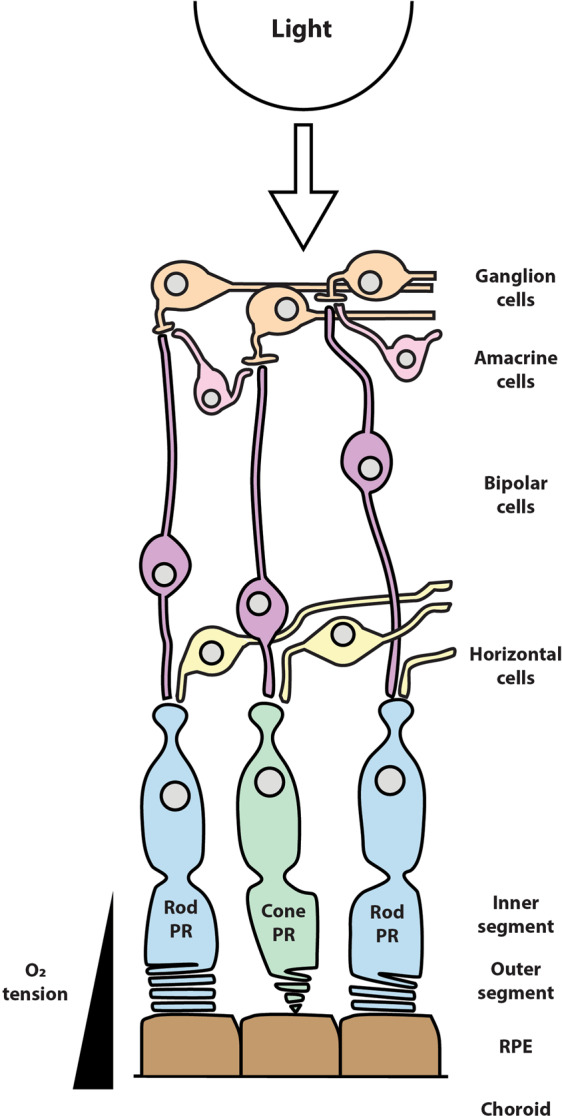
The laminated retina. Schematic representation of the retina and the direction of light. Photoreceptors are the outer most layer of the neural retina and include an inner segment, which contains the majority of the mitochondria, and an outer segment, where phototransduction occurs via rhodopsin and cone opsins. Outer segments are shed with integration into nearby RPE. Oxygen tension from the choroid rapidly decreases to its lowest in the mitochondria-rich inner segment region.

The retina is an intricately organized neural tissue at the innermost aspect of the eye that contains photoreceptors (PRs), which are highly specialized neurons responsible for phototransduction (Fig. 1). PR dysfunction occurs from various causes and contributes to vision loss in many retinal disorders, including age-related macular degeneration (AMD), inherited retinal diseases (IRDs), and retinal detachment. AMD affects an estimated 200 million individuals worldwide and is the leading cause of blindness for the elderly in the developed world^1,2^. IRDs, including retinitis pigmentosa (RP), affect over 2 million individuals worldwide and are a heterogenous group of inherited disorders with over 280 identified genetic mutations^3–5^. Retinal detachment affects over 850,000 people worldwide^6–8^. The vision loss patients experience secondary to these retinal disorders results in reduced quality of life, life-long clinical care, and loss of productivity for patients and caregivers^9^. Currently, there is only one Food and Drug Administration-approved treatment for IRDs and is efficacious only for a single IRD-causing mutation^10^. However, given the hundreds of mutations that cause IRDs, gene therapy is a limited therapeutic option and is expected to target a fraction of IRDs in the foreseeable future. Despite efforts to develop new AMD therapies, many, including the complement inhibitor lampalizumab, have failed to demonstrate efficacy^11^. There is an urgent unmet need for neuroprotective therapies that improve PR survival and prevent vision loss.

The retina is one of the most energy-demanding tissues in the body, and this requirement is driven largely by the metabolic needs of PRs^12^. Systemic mutations of enzymes important in cellular metabolism have been associated with isolated retinal degenerations^13–15^, suggesting that precise control of metabolic homeostasis may be central to long-term PR survival^16,17^. Therefore, a more nuanced understanding of the various metabolic pathways that modulate PR survival is expected to reveal novel molecular targets for pan-disease neuroprotective therapies.

## Photoreceptor metabolic demands

The importance of cellular metabolism in PRs stems from their prodigious energy demands and biosynthetic requirements. PRs are exceptionally active neurons, maintaining phototransduction, neurotransmission, and biosynthesis for outer segment (OS) recycling^18^.

Rods and cones are the two main types of PRs that exist in the mammalian retina. While they share many characteristics, key differences in the metabolic demands of rods and cones may contribute to disease pathogenesis^19^. The OS accounts for most of the structural differences between cones and rods. Rods are cylindrical in shape and consist of enclosed membrane discs, while cones are conical and have a continuous membrane with a significantly larger surface area^20–22^. The rhodopsin pigment located in rods is well-geared toward low-light vision while the cone opsin pigments in cones require higher illumination to provide added acuity and color vision^23^. In primates, cones make up only 5% of the PR population and dominate the central fovea of the macula^24^. These structural and functional differences are expected to extend to unique metabolic demands for each PR type.

### Physiologic requirements

As reviewed by Hurley et al.^12^, PRs are directly or indirectly responsible for most of the metabolism of the retina^25^. This, contextualized to the exceptionally high metabolic activity of the retina relative to other tissues, underscores the incredible metabolic requirements of PRs. Most of the energy is required to power Na^+^/K^+^ ATPase ion channels to maintain appropriate ion concentrations for phototransduction^26^.

In contrast to most neurons, PRs are depolarized in darkness and respond to light by hyperpolarizing. This default depolarized state, termed the dark current, requires constant energy supply to power the Na^+^/K^+^ ATPase channels in PR inner segments (IS). Thus, PRs paradoxically require more energy than almost any other cell type in darkness^26,27^. As light triggers PRs, these ion channels close, which results in neuronal hyperpolarization and phototransduction^26,28^. Concurrently, metabolic demand falls by an estimated five-fold^12,26^.

The specialization of cones to high acuity vision makes them less sensitive to light than rods. Consequently, rods are more often hyperpolarized than cones despite the same light exposure^29^. Cones are suspected to have increased metabolic demands to maintain the default depolarized state^29,30^, which aligns with their higher glycogen stores^31^. Furthermore, changes in glucose level and inhibition of glycolysis affect rods more than cones, suggesting that rods may depend more on glycolytic metabolism than cones^32–34^. However, the metabolic differences between rods and cones have been investigated primarily in rod-dominant animal models, and more studies using cone-dominant experimental models are needed to verify these findings.

Phototransduction occurs in the PR OS, which consists of lipid and protein rich stacked membranes dedicated to light capture (Fig. 1). The OS is the primary site of light-induced oxidative damage and is segregated from the rest of the PR cell body. To address this oxidative damage, PRs shed ~10% of the OS daily to recycle damaged membranes^18^. Thus, PRs must continually regenerate OS membranes, requiring not only catabolic energy generation, but also anabolic production of lipids, proteins, and nucleic acids. Baseline physiologic requirements place an exceedingly high metabolic demand on PRs to generate the energy and macromolecules necessary for normal visual function and survival. Given their little reserve capacity in maintaining these processes, PRs are particularly vulnerable to metabolic perturbations^35^.

### Photoreceptors are highly sensitive to metabolic dysfunction

The importance of maintaining metabolic homeostasis in PRs is highlighted by the presence of isolated retinal degenerations due to mutations in enzymes critical for energy and purine metabolism in all tissues^13,14^. Disruption of glycolysis through mutations of hexokinase isoforms *HK1* and *HKDC1* results in RP (Table 1)^15,36^. Isocitrate dehydrogenase subunit beta (*IDH3B*) is important for the tricarboxylic acid (TCA) cycle and nicotinamide adenine dinucleotide (NAD+) biosynthesis. Importantly, patients homozygous for certain *IDH3B* mutations present with isolated, nonsyndromic RP and have no other systemic manifestations, even though these mutations affect metabolic/oxidative processes in many tissues^13^. Consistent with this finding, multiple mutations in *IDH3A* are also associated with nonsyndromic RP^37,38^. Mutations in phosphodiesterase 6 (*PDE6*) are estimated to be responsible for 5–8% of all RP cases^39–41^. These mutations disrupt the physiologic hydrolysis of cGMP to GMP^42^. Interestingly, purine synthesis has also been reported to be regulated by inosine monophosphate dehydrogenase 1 (*IMPDH1*) and the presence of light^43^, and mutations in *IMPDH1* result in nonsyndromic IRDs^14,43^. Isolated ophthalmic manifestations of these genetic mutations emphasize the importance of maintaining the outer retinal metabolic balance for PR survival. In light of these clinical findings, recent studies have begun to unravel outer retinal metabolism and demonstrate that reprogramming PR metabolism may be an inventive neuroprotective strategy in retinal degenerative diseases^16,17,44,45^.

**Table 1:** Metabolic genes involved in photoreceptor degeneration.

Gene	Name	Pathway	Species	Phenotype	References
*IMPDH1*	Inosine monophosphate dehydrogenase 1	Nucleotide biosynthesis	Human, mouse	Retinitis pigmentosa	^14,171,172^
*PDE6*	Phosphodiesterase 6	Nucleotide biosynthesis	Hhuman, dog, mouse	Retinitis pigmentosa	^39–41,173,174^
*HK1* *HKDC1*	Hexokinase 1, Hexokinase Domain-Containing 1	Glycolysis	Human	Retinitis pigmentosa	^15,36,175^
*Hk2*	Hexokinase 2	Glycolysis	Mouse	Photoreceptor degeneration	^47,82^
*Pkm2*	Pyruvate kinase muscle isozyme 2	Glycolysis	Mouse	Photoreceptor degeneration	^78,81,90^
*Ldha*	Lactate dehydrogenase A	Glycolysis	Mouse	Retinal outer segment shortening	^90^
*Glut1*	Glucose transporter 1	IPM ecosystem/glycolysis	Mouse	Retinal outer segment shortening and photoreceptor death	^34^
*Mct3*	Monocarboxylate transporter 3	IPM ecosystem/glycolysis	Mouse	Impaired neuroretinal signaling	^74^
*Bsg*	Basigin	IPM ecosystem/glycolysis	Mouse	Photoreceptor death and impaired neuroretinal signaling	^73^
*Pi3k*	Phosphoinositide 3-kinase	mTOR	Mouse	Photoreceptor death and impaired neuroretinal signaling	^95^
*Pten*	Phosphatase and tensin homolog	mTOR	Mouse	Improved neuroretinal signaling in retinitis pigmentosa	^96^
*Tsc1*	Tuberous sclerosis 1	mTOR	Mouse	AMD-like phenotype	^98^
*Sirt6*	Sirtuin 6	mTOR/NAD biosynthesis	Mouse	Improved photoreceptor survival in retinitis pigmentosa	^17^
*Pgc-1a*	PPARγ coactivator proliferator-activated receptor gamma coactivator 1 alpha	mTOR/Fatty acid oxidation	Mouse	AMD-like phenotype with high-fat diet	^139^
*VLDLR*	Very low-density lipoprotein receptor	Fatty acid oxidation	Human	Macular atrophy	^140^
*Vldlr*	Very low-density lipoprotein receptor	Fatty acid oxidation	Mouse	AMD-like phenotype	^114^
*ACOX*	Acyl-CoA oxidase	Fatty acid oxidation	Human	Retinitis pigmentosa	^176,177^
*LCHAD*	Long chain L3 hydroxyacyl-CoA dehydrogenase	Fatty acid oxidation	Human	Macular pigmentary deposits and atrophy of choroid, RPE and retina	^178,179^
*Fatp4*	fatty acid transport protein 4	Fatty acid oxidation	Mouse	Light-induced photoreceptor degeneration	^132^
*ELOVL4*	Elongation of very long chain fatty acids 4	Fatty acid oxidation	Human, mouse	Inherited retinal degeneration, Stargardt disease	^180,181^
*Ppara* *Pparg*	Peroxisome proliferator-activated receptor	Fatty acid oxidation	Mouse	Decreased retinal vascular density	^135,136^
*Mpc1*	Mitochondrial pyruvate carrier 1	TCA Cycle	Mouse	Photoreceptor loss and impaired neuroretinal signaling	^117^
*IDH3A* *IDH3B*	Isocitrate dehydrogenase subunit alpha and beta	TCA Cycle/NAD metabolism	Human	Retinitis Pigmentosa	^13,37,38^
*Idh3a*	Isocitrate dehydrogenase subunit alpha	TCA Cycle/NAD metabolism	Mouse	Photoreceptor loss and impaired neuroretinal signaling	^182^
*Nampt*	Nicotinamide phosphoribosyltransferase	NAD biosynthesis	Mouse	Photoreceptor loss, pigment mottling, RPE atrophy, and impaired neuroretinal signaling	^108^
*NMNAT1*	Nicotinamide mononucleotide adenylyltransferase 1	NAD biosynthesis	Human, mouse	Macular colobomas, Leber congenital amaurosis	^183–185^
*Sarm1*	Sterile alpha and Toll/interleukin-1 receptor motif-containing 1	NAD biosynthesis/Redox homeostasis	Mouse	Improved photoreceptor function and survival	^165^
*Gpx4*	Glutathione-peroxidase	Redox homeostasis	Mouse	Photoreceptor loss	^155^

Gene symbols are listed here in accordance with the species. If mutations in the same gene have been investigated in different species, the genes are either grouped together (in the same row) if they share the same retinal phenotype or are listed apart if they have different phenotypes. In the case where mutations in the same gene are grouped together in a row due to shared phenotype, the symbol defers to the human nomenclature.

## Photoreceptor aerobic glycolysis

Glucose has been central in the study of PR metabolism as unlike most other neurons, PRs perform aerobic glycolysis^46–48^, or the conversion of glucose to lactate despite the presence of oxygen^49,50^. Studies have demonstrated that 80–90% of the glucose delivered to the outer retina is converted to lactate via aerobic glycolysis^51^. PRs may employ this unique metabolic adaptation to meet their high demand for energy and biosynthetic intermediates, similar to cancer cells^47,50,51^. At first glance, aerobic glycolysis appears to be a peculiar strategy for PRs to generate the ATP necessary to meet their high metabolic demands. The two ATP molecules generated from glycolysis pales in comparison to the theoretical 38 ATP molecules produced with completion of cellular respiration^52,53^.

**Fig. 2:**
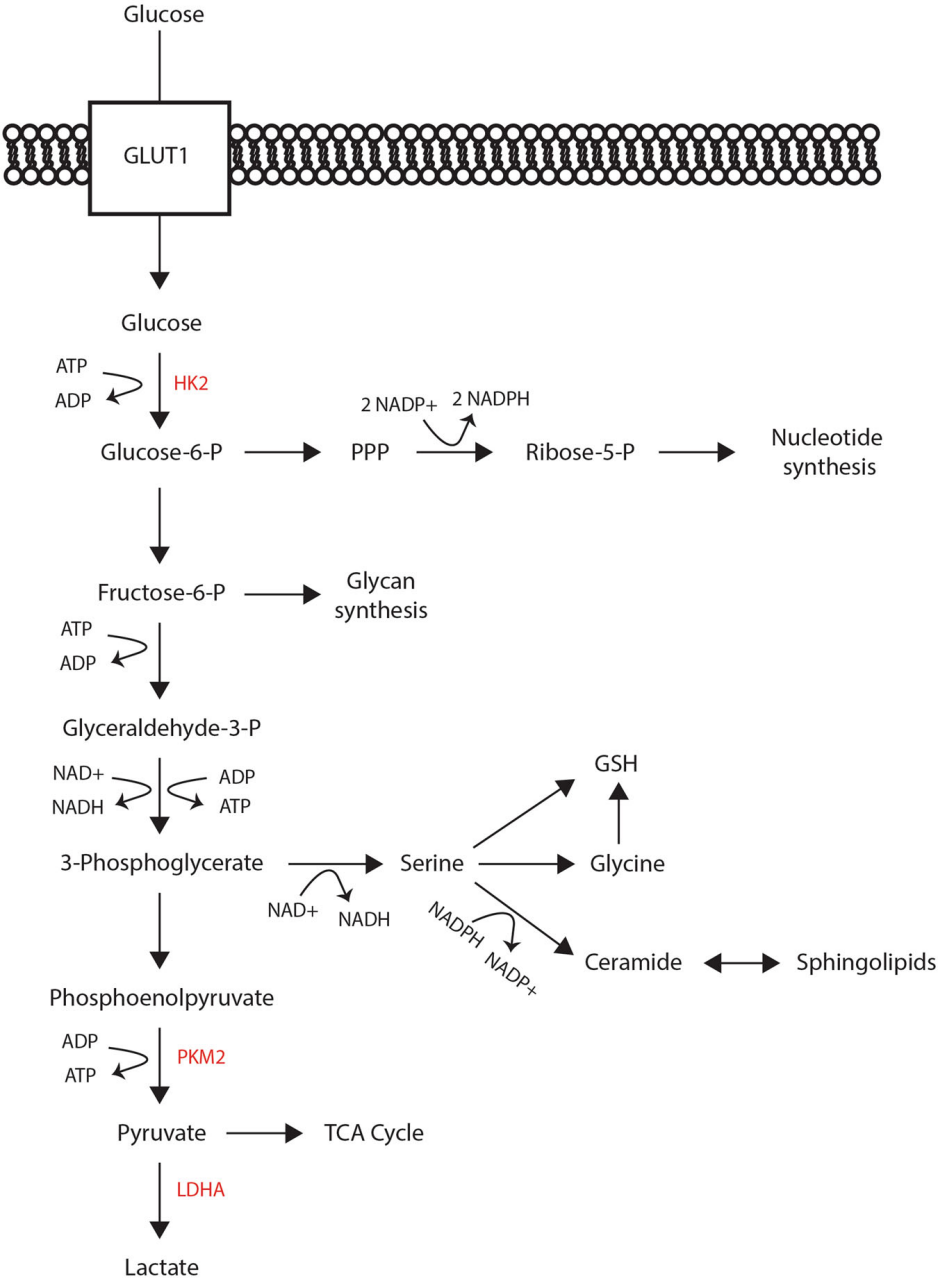
Aerobic glycolysis. The molecular steps of aerobic glycolysis and the branching pathways of the pentose phosphate pathway (PPP), glycan synthesis, and serine biosynthesis. Mutated or deleted glycolytic enzymes associated with degenerative retinal phenotypes are highlighted in red (i.e., HK2, PKM2, and LDHA). Details and references for these mutations are specified in Table 1. Nicotinamide adenine dinucleotide and nicotinamide adenine dinucleotide phosphate in their reduced (NADH and NADPH) and oxidized (NAD+ and NADP+) forms and adenosine diphosphate (ADP) and adenosine triphosphate (ATP) are detailed. Glucose-6-P glucose-6-phosphate, fructose-6-P fructose-6-phosphate, glyceraldhyde-3-P glyceraldehyde 3-phosphate, GSH glutathione, HK2 hexokinase 2, PKM2 pyruvate kinase muscle isoform 2, and LDHA lactate dehydrogenase A.

Aside from ATP generation, however, preferentially utilizing aerobic glycolysis has distinct advantages. One key benefit is the faster kinetics for rapid generation of energy and carbon intermediates. These intermediates are the substrates for glycan synthesis, the pentose phosphate pathway (PPP), and serine production pathway (Fig. 2). Glycans are involved in a variety of biochemical functions, including the glycosylation of rhodopsin, without which PRs degenerate^54,55^. The PPP is critical for anabolic synthesis of macromolecules like ribose and the generation of nicotinamide adenine dinucleotide phosphate (NADPH), which is involved in combating oxidative stress. Serine biosynthesis in the retina was recently reviewed^56^ and contributes to the synthesis of NADH/NADPH, sphingolipids, glutathione (GSH), and glycine. Importantly, macular telangiectasia type 2 and autosomal recessive retinitis pigmentosa have been genetically associated with disruptions in multiple steps of serine and glycine metabolism^57–59^. Given the huge anabolic load PRs engage in every day and the intense oxidative stress they are exposed to, the formation of serine, with its downstream creation of membrane materials (i.e., sphingolipids and ceramide) and antioxidants (i.e., NADPH and GSH), is significant. The inhibition of glycolysis and disruption of serine metabolism result in impaired OS structure and function^60^. Thus, despite the forfeit of ATP offered through oxidative phosphorylation (OXPHOS), aerobic glycolysis provides fundamental advantages in kinetics, intermediates for OS biosynthesis, and reduction-oxidation (redox) balance. Furthermore, aerobic glycolysis plays an important role in the metabolic ecosystem of the outer retina.

**Fig. 3:**
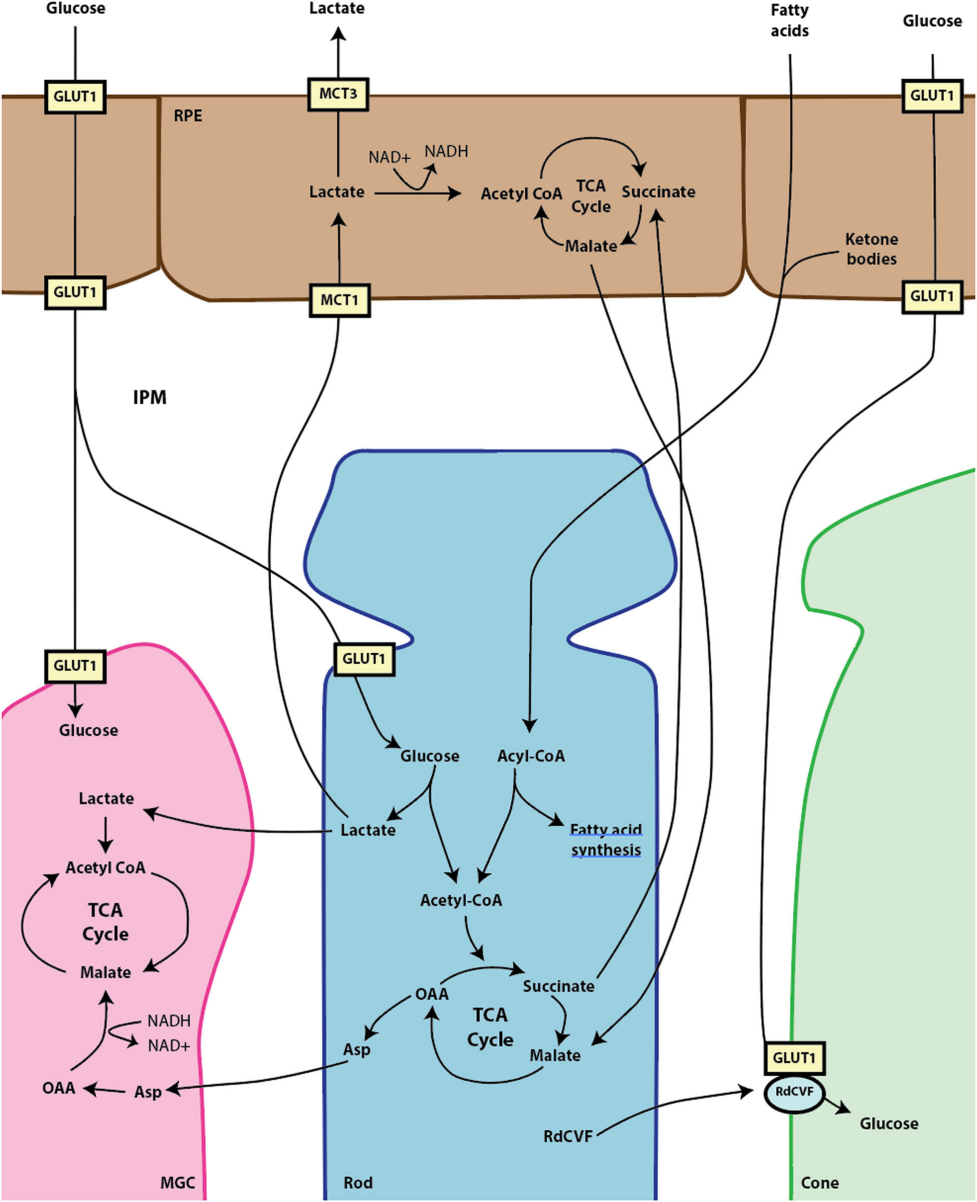
The outer retinal metabolic ecosystem. Glucose from the choroid enters and exits the RPE (retinal pigment epithelium) via GLUT1 (glucose transporter 1) to enter MGCs (Müller glial cells) and rod and cone photoreceptors via GLUT1. Rods secrete RdCVF (rod-derived cone viability factor), which complexes with GLUT1 in cones and enhances glucose entry. Glucose then either undergoes aerobic glycolysis to produce lactate or enters the TCA cycle to produce metabolic intermediates like aspartate and succinate. Lactate enters the interphotoreceptor matrix (IPM), proceeds to the nearby MGC and RPE via MCT1 (monocarboxylate transporter 1) and is either used as a substrate for the TCA cycle or exit the RPE into the choroid via MCT3 (monocarboxylate transporter 3). To regenerate the nicotinamide adenine dinucleotide (NAD+) necessary for lactate-driven respiration, aspartate is converted to OAA (oxaloacetate) and subsequently, malate to enter the TCA cycle in the MGCs. In PRs, fumarate can also be reduced to succinate, transported to RPE, and enter the TCA cycle. In return, RPE sends malate back to PRs. Additionally, fatty acids from the choroid and ketone bodies from the RPE enter photoreceptors, where they are converted to acyl-CoA and enter the TCA cycle or undergo fatty acid synthesis.

The PR OS abuts the apical surface of RPE, which forms the outer blood retinal barrier via tight junctions (Fig. 3). The basolateral surface of the RPE is adjacent to Bruch’s membrane and the choroidal vasculature, which perfuses the RPE and sclera^12^. Importantly, PRs, RPE, and Muller glia cells (MGCs) closely interact in the interphotoreceptor matrix (IPM) with exchange of macromolecules^12^. The outer retina metabolic ecosystem is critical for the survival and function of these cells.

PRs produce and secrete large amounts of lactate, which is taken up by neighboring RPE and MGCs and used as fuel. In RPE, pyruvate proceeds through the TCA cycle for ATP generation^61^. MGCs are even more dependent on PRs for lactate as an energy source since they lack the enzyme pyruvate kinase (PK) necessary for glycolysis^62^. Similar to RPE cells, MGCs use pyruvate to produce energy and metabolic intermediates through the TCA cycle and OXPHOS^62,63^. Reciprocally, the glutamine generated from the TCA cycle is transported from MGCs back to PRs to be used in metabolic and redox processes (Fig. 6)^62^.

With the PR-generated lactate acting as the metabolic fuel for the RPE, glucose is transported from the choroidal vasculature to PRs via the RPE (Fig. 3). Low expression of hexokinase (HK; a critical enzyme for glycolysis) and lactate’s ability to suppress glucose consumption in RPE^61,64–67^, promote the delivery of unmetabolized glucose to the PRs. In healthy outer retina, most of the glucose taken up from the choroidal circulation is used by PRs^61^. When the glycolytic flux and glucose metabolism in RPE are increased, PRs degenerate due to decreased glucose transportation and starvation^68,69^. These experimental findings are consistent with RP pathophysiology, where RPE increase glucose metabolism and restricted glucose transport to PRs result in retinal degeneration^64,70^.

The movement of glucose from the choroid to PRs is facilitated by a number of transporters. Glucose transporter 1 (GLUT1) importantly transports glucose from the choroid through the basolateral side of RPE, from the RPE through the apical side, and from the IPM into PRs (Fig. 3)^34,44^. Genetic deletion of *Glut1* in RPE results in PR degeneration^34^. Furthermore, an experimental model of RP exhibits decreased GLUT1 expression on the apical surface of RPE, thereby sequestering glucose in the RPE^70^. Intervention to restore GLUT1 to the apical side of the RPE improves neuroretinal signaling and visual function in this IRD model^70^.

Monocarboxylate transporters 1 (MCT1) and 3 (MCT3) facilitate movement of lactate from the IPM into RPE and from RPE into the choroid, respectively^71,72^. MCT1 and MCT4 are also expressed by rods and are thought to be necessary for promoting aerobic glycolysis by facilitating metabolite exchange in the outer retina^73^. Mice null for *Mct3* accumulate lactate in the retina and demonstrate impaired neuroretinal signaling^74^. Interestingly, the *MCT3* locus was found on GWAS to be associated with AMD and may contribute to disease pathogenesis^75^.

The retinal MCTs assemble with and require basigin (BSG) for lactate transport^71,73^. Genetic deletion of *Bsg* impairs neuroretinal signaling and causes PR degeneration by altering outer retinal lactate homeostasis^73,76^. A number of conditional deletion experiments of *Bsg* found that its absence in rods results in profound rod degeneration and functional abnormalities, its absence in RPE causes mild PR degeneration and functional abnormalities, and its deletion in cones is largely inconsequential^73^. Together, these experiments demonstrate the dynamic outer retinal ecosystem, where the RPE supplies glucose to PRs, which reciprocally generate lactate via aerobic glycolysis to fuel RPE and MGCs, and disruption in the flow of macromolecules from inappropriate sequestration results in retinal degeneration.

### Critical enzymes in photoreceptor aerobic glycolysis

Hexokinase 2 (HK2), pyruvate kinase muscle isoform 2 (PKM2) and lactate dehydrogenase A (LDHA) are critical isoforms that regulate aerobic glycolysis and the production of lactate by PRs. Recent studies have shown that these essential regulatory enzymes are important for the biosynthetic fidelity, function, and survival of PRs under physiologic conditions and during periods of outer retinal stress^47,77–82^.

HK catalyzes the first step in glycolysis (Fig. 2). Of the four isoforms, HK1 is the most widely expressed and HK2 is present in cancer cells and PRs, which coincides with their predilection for aerobic glycolysis^44,47,82,83^. In cancer, HK2 preferentially funnels glucose into aerobic glycolysis^77,84^. Petit et al. showed that rod PR-specific deletion of *Hk2* results in decreased aerobic glycolysis and increased OXPHOS^47^. However, a recent report from our laboratory did not show significant alterations in aerobic glycolysis in the retinas of a similar transgenic mouse model^82^. Despite these metabolic differences, both reports demonstrate that PRs need HK2 for function and adaptation to metabolic stress^47,82^. Hence, the nonmetabolic functions of HK2 may also be playing a role in the preservation of PRs during outer retinal stress. HK2 normally resides on the mitochondria where it receives preferential access to ATP for the phosphorylation of glucose to glucose-6-phosphate. While localized to the mitochondria, HK2 also prevents the binding of proapoptotic factors to the mitochondria and the resultant apoptotic signaling. PRs are constantly under oxidative stress in part due to the free radicals produced in the OS by light, and Rajala et al. observed that light regulates the binding of HK2 to the mitochondria^85^. Additionally, we reported that in the presence of acute metabolic stress secondary to experimental retina-RPE separation, HK2 translocates to the mitochondria and conditional deletion of HK2 in rod PRs increases their susceptibility to apoptosis^82^. Therefore, understanding the exact metabolic and nonmetabolic functions of HK2 in PRs has the potential to reveal novel strategies for PR neuroprotection.

Pyruvate kinase (PK) catalyzes the final rate-limiting step of glycolysis by converting phosphoenolpyruvate (PEP) and adenosine diphosphate (ADP) to pyruvate and ATP (Fig. 2). Similar to cancer cells, terminally differentiated PRs maintain expression of the PKM2 isoform to perform aerobic glycolysis^86^, even though most other neurons exclusively express PKM1 after maturation to maximize OXPHOS capacity^62,87^. In contrast to the constitutively active PKM1 isoform, PKM2 activity is tightly regulated. In its tetrameric form, PKM2 is maximally active and associated with increased catabolic ATP synthesis. Tetrameric state is favored by nutrient deprivation and certain allosteric regulators, including serine and fructose-1,6-bisphosphate^79,88^. In contrast, dimeric PKM2 has lower catalytic activity and is associated with increased anabolic biosynthesis pathways. This catalytic state is supported by nutrient excess, growth factors, and post-translational modifications, including phosphorylation^89^. Shifting between distinct quaternary structures may allow PKM2 to regulate metabolic budgeting and balance anabolic and catabolic responses to match the metabolic needs of PRs.

Recent studies have examined the importance of PKM1 and PKM2 in PR metabolism. Using in vivo electroporation, Chinchore et al. demonstrated that OS maintenance requires PKM2, and that PKM1 isoform substitution is unable to rescue diminished OS length^90^. Our laboratory and others have used a mouse model where conditional deletion of *Pkm2* in rod PRs results in compensatory expression of *Pkm1*^78,81^. This isoform switch leads to an increase in glycolytic intermediates and a small but significant decrease in rod OS biogenesis, function, and survival. Similar structural and functional changes have been observed in cone PRs after PKM2 deletion^80^. Interestingly, PKM2-to-PKM1 isoform switch boosts rod PR survival during acute nutrient stress secondary to experimental retinal detachment^78^. In wild-type mice, experimental retinal detachment decreases PKM2 tyrosine phosphorylation, which promotes dimer to tetramer transition and increases catalytic activity^78^. Therefore, the PKM2-to-PKM1 isoform switch observed in PR-specific deletions of *Pkm2* may mimic the increase in catalytic activity by substituting constitutively active PKM1 for tetrameric PKM2. Similarly, we demonstrated that ML-265, a small molecule activator of PKM2 that stabilizes the tetrameric form, reduces PR entrance into the apoptotic cascade in in vitro and in vivo models of outer retinal stress^79^. Thus, PKM2 modulation represents a novel therapeutic strategy to reprogram metabolism and improve PR survival in a multitude of retinal disorders. In support of this therapeutic paradigm, a recent publication suggests that PKM2 metabolic reprogramming may improve PR function and survival in an IRD mouse model^91^.

In the final step of aerobic glycolysis, LDHA converts pyruvate to lactate, which can be shuttled to neighboring RPE and MGCs as discussed above (Figs. 2, 3). Similar to HK2 and PKM2, LDHA is preferentially expressed in PRs in accordance with the location of aerobic glycolysis within the retina^50,90,92^. Similar to *Pkm2* knockdown, reducing *Ldha* expression via in vivo electroporation results in reduced OS length^90^. These studies from multiple research groups provide ample evidence that aerobic glycolysis and its key enzymes are important for maintaining the biosynthetic demands as well as the function and survival of PRs.

### Metabolic reprogramming of the glycolytic machinery

**Fig. 4:**
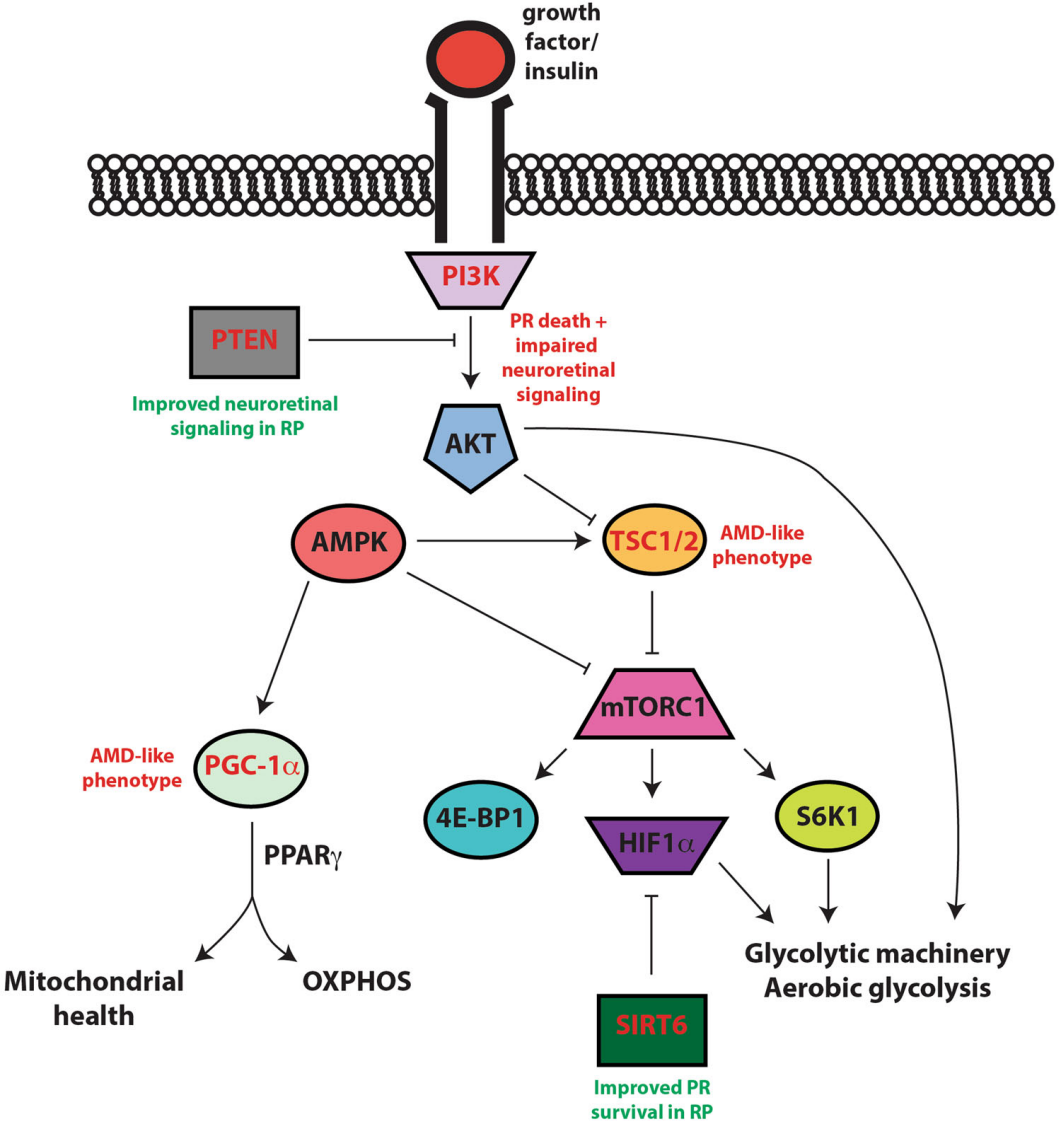
mTOR pathway in photoreceptors. This cartoon summarizes the proteins in the mTOR pathway that have been shown to be important in photoreceptor survival. Upon growth factor or insulin binding, PI3K (phosphosinositide 3-kinase) promotes AKT activation, which by inhibiting the inhibitor TSC1/2 (tuberous sclerosis complex protein 1/2) complex, activates mTORC1 (mammalian target of rapamycin complex 1) and stimulates eukaryotic translation initiation factor 4E-binding protein 1 (4E-BP1), ribosomal protein S6 kinase beta-1 (S6K1), and hypoxia-inducible factor 1 alpha (HIF-1α). S6K1 and HIF-1α increase glycolytic machinery and aerobic glycolysis^17,45^. Sirtuin 6 (SIRT6) also inhibits HIF-1α^17^. PTEN (phosphatase and tension homolog deleted on chromosome ten) inhibits PI3K-mediated activation of AKT, which also directly regulates key glycolytic machinery^82,85^ or controls transcription factors that promote the expression aerobic glycolysis components^186^. The nutrient sensor adenosine monophosphate activated protein kinase (AMPK) decreases mTOR signaling by activating TSC1/2 and inhibiting mTORC1 and activates PGC-1α (PPARγ coactivator proliferator-activated receptor gamma coactivator 1 alpha), to promote oxidative phosphorylation (OXPHOS) and mitochondrial health in conjunction with PPARγ. Genes mutated or deleted from PRs involved in this pathway are detailed in red (PTEN, PI3K, SIRT6, TSC1, and PGC-1α) with their associated retinal phenotypes nearby in red if neurodegenerative and green if neuroprotective. These specific mutations and references are detailed in Table 1.

In addition to specifically targeting the enzymes critical for aerobic glycolysis, an improved understanding of the importance of aerobic glycolysis as a whole in the fidelity of PRs has broadened the possible metabolic reprogramming strategies. Multiple research groups have identified mammalian target of rapamycin (mTOR) pathway-related targets as ways to reprogram the glycolytic machinery in PRs^16,17,45^. The mTOR pathway is involved in regulating cellular growth and metabolism, and is implicated in the pathogenesis of cancer and neurodegeneration, including RP^16,45,93^. mTOR, as a member of the phosphoinositide 3-kinase (PI3K) related kinase family, combines with other proteins to form the mTOR complex 1 (mTORC1), and is part of the downstream signaling pathway of growth factor activity (Fig. 4). In rodent models, PR degeneration is observed when mTOR, protein kinase B (AKT), or PI3K are inhibited or knocked out^16,82,94,95^. Additionally, a number of other studies demonstrate that preclinical models of IRD have decreased mTOR signaling, including increased PTEN (phosphatase and tension homolog deleted on chromosome ten) levels and decreased levels of phosphorylated AKT and mTOR, their activated forms^96^. A recent finding in our laboratory further linked phosphorylated AKT with HK2 translocation to the mitochondria and improved PR survival after retinal detachment^82^. Together, these findings support the hypothesis that decreased mTOR signaling drives PR degeneration.

In accordance with these findings, experimental enhancement of mTOR signaling in PRs has been shown to be neuroprotective^16,17,45^. General activation or suppression of mTOR activity through injections of insulin or streptozotocin, respectively, result in improved or worsened cone survival^16^. Constitutive activation of mTORC1 by genetically deleting inhibitors *Pten* and tuberous sclerosis complex protein 1 (*Tsc1*) enhances survival and function of cones in retinal degeneration models^45^. Similarly, augmentation of AKT activity protects rod and cone structural and functional integrity in RP models^97^. Neuroprotection via mTOR and AKT activation is attributed to improved glucose uptake, retention, and utilization by PRs^45^. This is consistent with the hypothesis that enhanced nutrient availability may be an effective strategy for boosting PR survival. In contrast, rod and cone-specific genetic deletion of *Tsc1* results in an AMD-like phenotype, suggesting that chronic augmentation of mTOR signaling may contribute to PR death^98^. The authors suggest that this may stem from increased lipid accumulation^98^. These divergent findings may be context dependent and require further studies to reveal the exact role of mTOR signaling in the outer retina.

These studies spurred examination into downstream targets of mTORC1 that may mediate PR neuroprotective effects. While ribosomal protein S6 kinase beta-1 (S6K1) and eukaryotic translation initiation factor 4E-binding protein 1 (4E-BP1) are both downstream (Fig. 4), only S6K1 activity was impaired with increased PTEN signaling (i.e., model of neurodegeneration) in RP mice^96^. Moreover, knockdown of S6K1 results in apoptosis of cones but knockdown of 4E-BP1 does not^96^. Expectedly, overexpression of S6K1 increases rod and cone survival in preclinical models of retinal degeneration^96^.

Adenosine monophosphate activated protein kinase (AMPK) is an energy sensor important in the regulation of metabolic homeostasis^99^. AMPK function is partly mediated through the mTOR signaling pathway and glycolysis. Metformin, an activator of AMPK, protects PR structure and function in multiple models of acute and chronic retinal degeneration^100^. This neuroprotection is attributed to the role AMPK plays in mitigating oxidative stress^100,101^, promoting mitochondrial biogenesis, and increasing ATP levels through glycolysis^100^.

Similar to the multiple neuroprotective strategies targeting glycolysis, rod-derived cone viability factor (RdCVF), an endogenous protein secreted by rods to bolster glucose uptake by neighboring cones, acts as a cone survival factor in IRD models (Fig. 3)^44^. RdCVF joins with GLUT1 and BSG1 to create a complex on cone surfaces that enhances glucose uptake and consequently aerobic glycolysis^44^. In various models of IRD, RdCVF protects cones at the exclusion of rods, thereby explaining the human progression of disease: peripheral night vision loss (i.e., rod death) that is ultimately followed by central blindness (i.e., cone death)^44,102–104^. It is unclear why RdCVF only protects cones, but the suspected metabolic differences between rods and cones likely play a primary role. Therefore, increased cone aerobic glycolysis via RdCVF not only helps explain the manifestation of IRDs, but also represents a native mechanism that can be finessed to protect vision. SPVN06, an RdCVF replacement strategy, is currently being developed as an IRD gene therapy by SparingVision^105^.

Sirtuins are a group of NAD-dependent deacetylases involved in metabolic homeostasis. All seven mammalian sirtuins (SIRT1-7) are expressed in the retina^106^, and SIRT1, 3, and 5 have all been implicated in retinal degeneration^106–108^. Furthermore, SIRT6 is a histone H3K9 deacetylase involved in the mTOR pathway as a repressor of transcription factor hypoxia-inducible factor 1 alpha (Hif-1α)^109^. Hif-1α is an important transcriptional regulator of many proglycolytic genes and has been implicated in aerobic glycolysis for cancer cells^110^. Importantly, rod-specific deletion of *Sirt6* improves both rod and cone structure and function in experimental models of IRD via the upregulation of glycolytic metabolism (Fig. 4)^17^.

## Oxidative phosphorylation

The TCA cycle and OXPHOS both occur in the mitochondria. In humans, the vast majority of PR mitochondria are located in the IS^111^, where oxygen tension is at its relative lowest^112^, suggesting that oxygen is constantly being used by PRs for respiration (Fig. 1). Interestingly, the amount of oxygen used by rods (via a rabbit model) and cones (via a porcine model) is significantly higher in darkness, pointing to the importance of OXPHOS for dark current maintenance^25,113^. In these same studies, lactate levels are unchanged, suggesting that levels of aerobic glycolysis are constant in light and dark^25,113^. While aerobic glycolysis is used by PRs and is the pathway by which the vast majority of glucose is metabolized^114^, the less than 20% of glucose that enters OXPHOS has been proposed to account for 80% of the total ATP generation^51,113,115,116^. Genetic disruption of mitochondrial pyruvate carrier 1 *(Mpc1)* prevents pyruvate transport into the mitochondria, effectively inhibiting the TCA cycle, and results in PR degeneration^117^. In addition to impaired OXPHOS, this study demonstrated the importance of the TCA cycle as a source of biosynthetic intermediates and the flexibility of PRs in utilizing alternative fuel sources, including glutamine and β-hydroxybutyrate, to maintain the TCA cycle^117^. A major caveat in these experiments is the pan-retinal disruption of *Mpc1*, and the possibility that observed PR death may not be cell autonomous^117^.

When oxygen is not available to PRs, the TCA intermediate fumarate may substitute as an electron acceptor. Fumarate is converted to succinate via reversal of the TCA cycle and shuttled into nearby RPE, where oxygen tension is higher and OXPHOS can be completed^118^. In return, RPE provides malate back to PRs, supplying the intermediate necessary for succinate generation^118^. While these experiments were performed ex vivo, they demonstrate the potential of the PR-RPE succinate shuttle in distributing TCA intermediates and reducing power between cell-types in the outer retina ecosystem. The malate-aspartate shuttle (MAS) is another key macromolecule exchange pathway in the outer retina^62^. Aspartate from PRs provide the oxidative power to convert NADH to NAD+, which is needed for neighboring MGCs to consume lactate to fuel the TCA cycle and mitochondria (Fig. 3)^62^. Other studies have demonstrated that the TCA intermediates derived from proline utilization in the RPE are transported to nearby PRs, which may contribute to the partial PR rescue by a proline-rich diet in a retinal degeneration mouse model^119^.

The capacity for OXPHOS is different between rods and cones^111^. Because cones have been shown to possess more mitochondria than rods^111^, investigators have pointed to this difference as possibly why *Hk2* deletion in rods, but not in cones, results in a compensatory increase in OXPHOS machinery^47^. Other studies examining calcium-induced PR degeneration as a model for RP discovered that rods undergo apoptosis when overloaded with calcium^120^, but cones are resistant^121^. Instead, increased calcium influx into cone mitochondria enhances glutamine catabolism in the TCA cycle^121^. These studies illustrate instances where structural differences have expanded into metabolic specialization in rods and cones.

## Lipid metabolism

Lipids in the form of polyunsaturated fatty acids (PUFAs) make up a significant proportion of the PR OS. Up to 10% of the OS is shed daily, which is phagocytosed and metabolized by the neighboring RPE^122–124^, delivering lipids back to PRs^124^. Lipid metabolism is important for the biosynthetic fidelity of PRs in both their anabolic construction of OS and metabolism for energy and intermediates. Dysfunction in this outer retinal ecosystem and lipid metabolism has been implicated in the pathogenesis of AMD^98,125^. AMD is characterized by drusen (extracellular lipid-rich debris) deposits on the apical and basolateral sides of RPE^126^. Advanced AMD manifests with RPE degeneration and the loss of overlying PRs, producing central vision impairment.

**Fig. 5:**
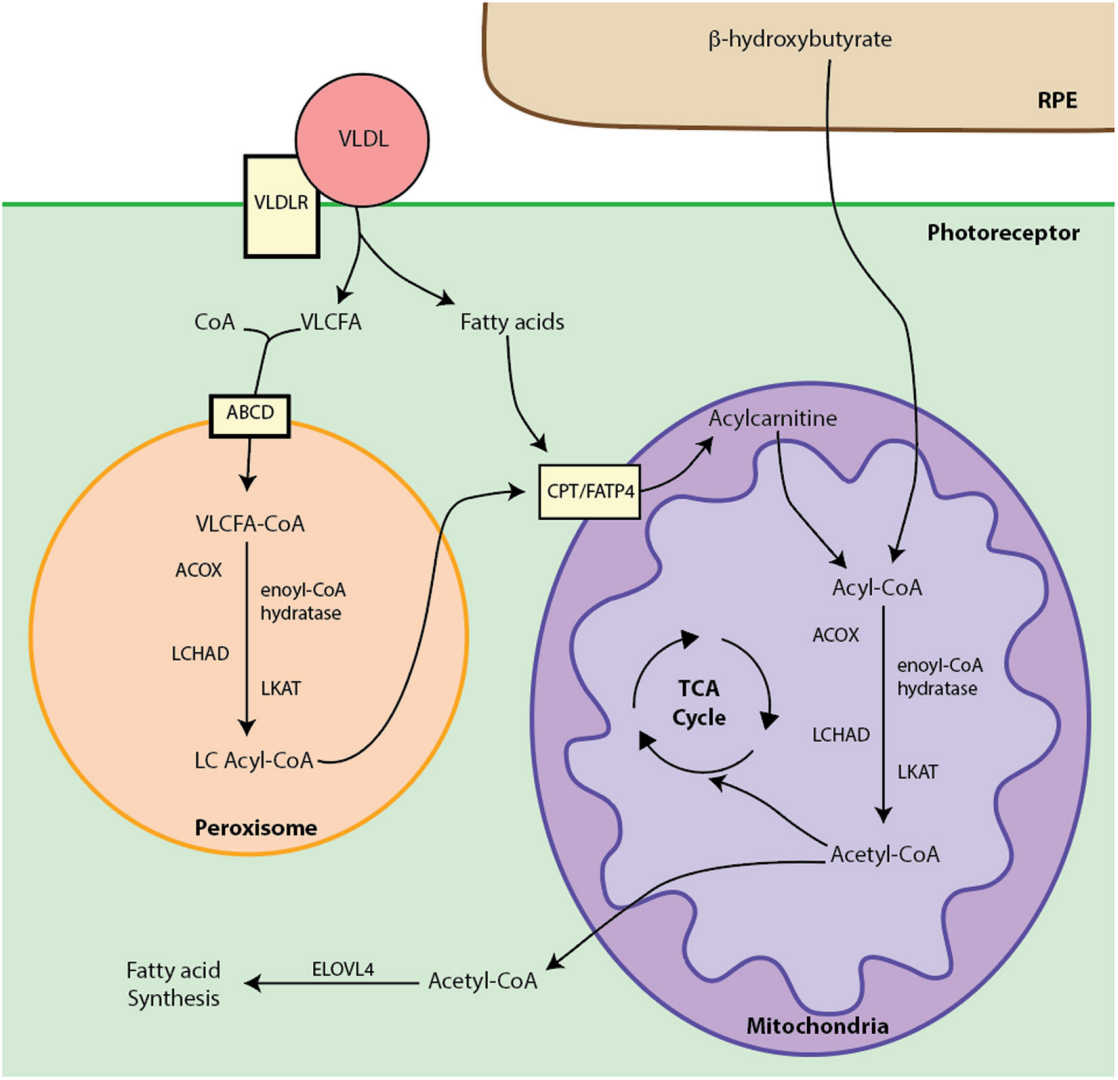
Fatty acid oxidation. The VLDLR (very low-density lipoprotein receptor) on photoreceptors facilitates binding of VLDLs (very low-density lipoproteins) and influx of VLCFAs (very long chain fatty acids; >22 carbons). VLCFAs join with CoA (coenzyme A) and enter peroxisomes where they undergo fatty acid β-oxidation (FAO) via acyl-CoA oxidase (ACOX), enoyl-CoA hydratase, LCHAD (long chain L3 hydroxyacyl-CoA dehydrogenase), and LKAT (long chain 3 ketoacyl-CoA thiolase). Processed LC acyl-CoA (long chain acyl-coenzyme A) proceeds to the mitochondria with other FAs via CPT (carnitine-palmitoyl transferase) and FATP4 (fatty acid transport protein 4). These FA derivatives, along with β-hydroxybutyrate from RPE enter the mitochondrial matrix as acyl-CoA, which is processed through FAO via enzymes ACAD (acyl-Coa dehydrogenase), enoyl-CoA hydratase, LCHAD, and LKAT. The acetyl-CoA produced can enter the TCA cycle or contribute to FA synthesis, which involves ELOVL4 (elongation of very long chain fatty acids 4).

The fatty acids (FAs) from the RPE^127^, peroxisome^124,128^, and cytosol enter the mitochondria and undergo fatty acid β-oxidation (FAO) to produce acetyl-CoA, flavin adenine dinucleotide (FADH_2_), and NADH, which subsequently contribute to anabolic FA synthesis or catabolic ATP generation (Fig. 5)^129^. Lipids are a substantial source of energy, as complete metabolism of a 16-carbon fatty acid produces a theoretical 129 ATP compared to the 38 ATP and 2 ATP from glucose in OXPHOS and glycolysis, respectively^129^. FAs have been hypothesized to be the substrate source for the unaccounted ATP generated by OXPHOS not originating from glucose in the rabbit retina^116^.

While still a newly explored area of research, very low-density lipoprotein receptor (VLDLR) has been shown to be important in PRs for the uptake of very long chain fatty acids (VLCFAs) from circulating chylomicrons (Fig. 5)^114^. Deletion and knockdown of *Vldlr* in PRs result in AMD-like retinal vascular abnormalities, decreased ATP generation, and pathologic structural changes in mitochondria^114^. The FAs that enter PRs via VLDLR undergo peroxisomal and mitochondrial FAO, which involves a number of enzymes including acyl-CoA oxidase, enoyl-CoA hydratase, long chain L3 hydroxyacyl-CoA dehydrogenase, and long chain 3 ketoacyl-CoA thiolase (Fig. 5). Mutations in these enzymes cause retinopathy^130,131^. In addition, mutation of the mitochondrial transporters carnitine-palmitoyl transferase and fatty acid transport protein 4 also results in retinal degeneration^132^. This even applies to the FA synthesis enzyme elongation of very long chain fatty acids 4 (ELOVL4), which when mutated causes a common form of inherited retinal degeneration, Stargardt disease^133^. These mutation-induced deficits in lipid metabolism appear to affect glucose metabolism in PRs, as the absence of VLDLR decreases both lipid and glucose metabolism in PRs^114^. The overlap between lipid and glucose metabolism is further supported by the observed decrease in genes involved in lipid metabolism with experimental disruption of aerobic glycolysis through *Pkm2* deletion^80,81^. Interestingly, *Pkm2* deletion does not alter the anabolic synthesis of phospholipids^80,81^.

Peroxisome proliferator-activated receptor (PPAR) alpha and gamma are nuclear receptor proteins involved in regulating lipid metabolism in the peroxisome and mitochondria^134^. The genetic deletion of *Ppara* and *Pparg* in mice results in impaired FA metabolism and retinal abnormalities^135,136^. Conversely, the use of PPARα agonist fenofibrate improves PR health in *Vldlr* null rodents, possibly secondary to improved mitochondrial FAO^135^. PGC-1α (PPARγ coactivator proliferator-activated receptor gamma coactivator 1 alpha) is an important intersection in AMPK-regulated glucose metabolism and mitochondrial lipid metabolism (Fig. 4)^101,137,138^. Consistent with these important roles, mice heterozygous for *Pgc-1α* develop PR degeneration^139^. This was thought to be a consequence of both decreased energy production and increased oxidative stress, which was corroborated with a study that pharmacologically activated PGC-1α^139^. While this area of investigation is still in its nascent stage, there are a number of retinal degenerations due to lipid dysfunction that make it an exciting avenue of research (Table 1)^130,140^.

Some of the retinal degenerative disorders due to peroxisome mutations have recently been reviewed, including Zellweger syndrome and adrenoleukodystrophy^130^. A particularly interesting finding is the successively decreased levels of VLCFAs in aged eyes and AMD eyes when compared to younger control eyes^141^. Moreover, human mutation of *VLDLR* results in retinal degeneration^140^. Also, mutations that disrupt VLCFA metabolism or PUFA synthesis have been shown to cause retinal degeneration^131,133,142,143^. These distinct retinal degenerations associated with dysfunction of lipid metabolism highlight the need to better understand lipid metabolic pathways in PRs and develop a framework for novel neuroprotective strategies targeting retinal lipid homeostasis.

## Oxidative stress

**Fig. 6:**
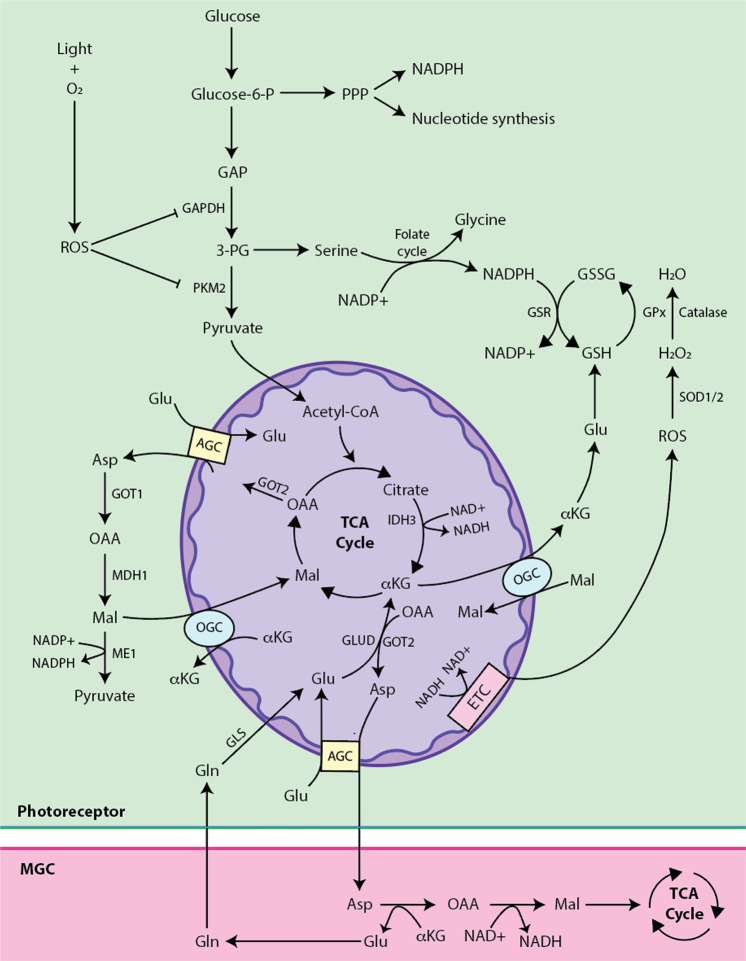
Redox metabolism in the outer retina. Exposure to light and oxygen and oxidative phosphorylation via the ETC (electron transport chain) generates ROS (reactive oxygen species) that can be reduced via NADPH, GSH (glutathione), and antioxidant enzyme systems. NADPH is a key reducing equivalent that can be generated by the PPP (pentose phosphate pathway), serine metabolism, and ME1 (malic enzyme 1). NADPH reduces GSSG via GSR (glutathione reductase) to GSH, which is generated from serine metabolism and Glu (glutamate). GSH powers the enzyme system GPx (glutathione-peroxidase), which along with the other systems catalase and SOD1/2 (superoxide dismutase 1 and 2) helps reduce ROS. Nearby MGCs can provide Gln (glutamine) to photoreceptors, where it is converted to Glu and enters the TCA cycle or shunted out of the mitochondria as αKG (alpha ketoglutarate) via OGC (oxoglutarate carrier). IDH3 (isocitrate dehydrogenase 3), GAPDH (glyceraldehyde 3-phosphate dehydrogenase), PKM2 (pyruvate kinase muscle isoform 2), H_2_O_2_ (hydrogen peroxide), Glucose-6-P (glucose-6-phosphate), GAP (glyceraldehyde 3-phosphate), 3PG (3-phosphoglyceric acid), GLUD (glutamate dehydrogenase), GOT2 (glutamic-oxaloacetic transaminase 2), OAA (oxaloacetate), Asp (aspartate), AGC (aspartate-glutamate carrier).

The retina, especially at the level of PRs, is particularly vulnerable to oxidative stress due to the high metabolism, oxygen, and light exposure^114,115,144^. The fundamental sequence in the visual cycle where light strikes rhodopsin and isomerizes 11-*cis*-retinal to all-*trans*-retinal creates significant numbers of reactive oxygen species (ROS) that cause lipid peroxidation of PUFAs in the OS^145^. Reduction mechanisms are in place to combat this overwhelming oxidation load in the form of NADPH, GSH, and antioxidant enzyme systems including glutathione-peroxidase (GPx), superoxide dismutase (SOD) and catalase (Fig. 6). The importance of these antioxidant enzymes in limiting oxidative damage has been comprehensively reviewed elsewhere^56,146,147^.

NADPH is a major cellular redox currency and is generated from the pentose phosphate, serine synthesis, and mitochondria-involved pathways (Figs. 2, 6)^48,148^. Examination into light-induced oxidative stress in skin demonstrates that the glycolytic machinery reroutes glucose intermediates into the PPP^149^ to promote NADPH generation and de novo nucleotide biosynthesis, countering ROS and DNA damage, respectively (Fig. 6)^149^. In PRs, the PPP has similarly been identified as a major source of NADPH during glucose replete states. Additionally, glutamine, pyruvate, and lactate are important in the generation of NADPH through the TCA cycle in the mitochondria^48,148^. The flexibility of PR metabolism to quickly transition from ATP generation to redox balance may be a unique metabolic adaption in oxidation-vulnerable PRs, which shuttle the majority of glucose to aerobic glycolysis^148,149^.

NADPH is also generated by malic enzyme 1 (ME1) from malate, IDH from isocitrate, and serine synthesis from 3-phosphoglycerate (Figs. 2, 6)^13,48^. Serine synthesis contributes to the production of serine, glycine, and cysteine, which are involved in the formation of GSH, along with anabolic macromolecules^150^. A recent review detailed the importance of serine metabolism in PR health^56^. Briefly, disruption of serine synthesis was found to significantly decrease FA synthesis, TCA intermediates, ATP production, and mitochondrial health^56^. These downstream metabolic effects may explain why many serine pathway mutations manifest as IRDs^56,151^.

GSH is produced when cysteine, as a consequence of serine biosynthesis or the cysteine-glutamate antiporter, combines with glutamate and is continually reduced by NADPH from its oxidized form, GSSG, by glutathione reductase (Fig. 6)^152^. Light-induced lipid peroxidation of PUFA, highly enriched in PR OS, requires the reductive potential of GSH and GPx to prevent PR cell death^153–155^. GSH levels are decreased in RP mice compared to controls, and GSSG levels increase after light exposure, supporting the notion that GSH combats light-induced oxidative damage^154^. The GPx4 isoform offers the most protection from lipid peroxidation by utilizing GSH and is crucial in the newly discovered cell death pathway of ferroptosis^153^. PR-specific deletion of *Gpx4* results in shortened OS, dysfunctional mitochondria, lipid peroxidation, and rod and cone cell death^155^. In contrast, PR-specific overexpression of *Gpx4* results in reduced oxidative damage and PR protection^156^. These studies suggest that GSH and GPx are critical components of the antioxidant system necessary for PR survival; therefore, the upkeep of glutamine concentration may be critical to maintain the redox balance in the outer retina.

Nearby MGCs and RPE provide much of the glutamine^62,157^, which when converted to glutamate via glutaminase (GLS) can either enter the catabolic TCA cycle or contribute to GSH generation (Fig. 6)^46^. Flux experiments demonstrate that 98% of glutamate in PRs is protected from catabolism in the TCA cycle due to the MAS^46^. Consistent with this, deletion of the aspartate-glutamate carrier 1 (*Agc1*) important in MAS results in decreased cytosolic glutamate concentrations^46^ and impaired light-evoked visual function^158^. Metabolic budgeting of glutamate between GSH synthesis and mitochondrial fuel is likely finely tuned in PRs. Retinal degeneration in *Mpc1* conditional knockout of mouse model may be a consequence of its reliance on glutamine as an fuel source in the absence of mitochondrial pyruvate and consequent depletion of glutamate as a substrate for GSH production^117^.

The antioxidant enzyme systems of SOD and catalase are also important in retinal redox homeostasis (Fig. 6). The SOD1 isoform, like GPx4, targets the cytosol and their joint overexpression in PRs protects cones by decreasing the oxidative damage experienced in a RP mouse model^159^. Coordinative overexpression of SOD2 and catalase, which both target the mitochondria, similarly preserves cones^160^. Yet, overexpression of these enzymes do not protect rods, providing further evidence for the metabolic differences between rods and cones^159,160^. Furthermore, these antioxidative and cone-specific protective effects are not observed when any of these four enzymes (i.e., SOD1, GPx4, SOD2, and catalase) are overexpressed alone or jointly with another enzyme that targets a different cellular compartment (e.g., cytosolic SOD1 with mitochondrial catalase)^159,160^.

As previously mentioned, AMPK exists at the intersection of glycolysis/OXPHOS metabolism and mitochondrial redox homeostasis (Fig. 4)^100^. Metformin-mediated AMPK stimulation protects PRs from light-induced damage and death^100^. This was attributed to the combination of protective redox mechanisms in elevated SOD2 and NADH/NAD+ ratio and the metabolic transition from ROS-generating OXPHOS to glycolysis^100^. Thus, AMPK signaling represents an example of the potential neuroprotective opportunity offered by reprogramming metabolism to achieve redox homeostasis (Figs. 4, 6).

NAD is a coenzyme for enzymes involved in redox reactions and a cosubstrate for other enzymes that regulate SIRTs^161^. NAD biosynthesis depends predominantly on the salvage pathway in mammals and requires nicotinamide phosphoribosyltransferase (NAMPT) as the first enzyme in this pathway^161^. This was recently reviewed by Lin and Apte^161^ and its importance in PR homeostasis corroborated by their discovery of low NAD+ levels in multiple experimental models of retinal degeneration^108^. Furthermore, the PR-specific genetic deletion of *Nampt*, results in structural and functional deficits in PRs^108^. Administration of nicotinamide mononucleotide (NMN), a NAD+ intermediate, in this *Nampt* null mouse and in a RP mouse model protects PRs^108,162^. Consistent with this, overexpression of *Nampt* increases NAD+ levels, boosts PR function, and restores extracellular NAMPT levels^163^. Furthermore, increasing NAD+ levels via overexpression of nicotinamide nucleotide adenylyltransferase 1 protein (*NMNAT*), which catalyzes NMN to NAD+ is neuroprotective^164,165^. Interestingly, sterile alpha and Toll/interleukin-1 receptor motif-containing 1 (SARM1) is a major regulator of neurodegeneration through the modulation of NAD+ concentrations^166,167^. SARM1 is not only expressed in PRs, but its activation also results in NAD depletion and PR death^165^. Additionally, its deletion is neuroprotective and rescues the NMNAT retinal degeneration phenotype^168^. These discoveries, coupled with the decreasing extracellular NAMPT levels humans experience with aging, suggest a possible connection between NAD+ and PR degeneration in RP and AMD^163^. To this end, patients with mutations in *IDH3B*, which encodes the β subunit of the NAD-specific isocitrate dehydrogenase (NAD-IDH), experience RP suggesting the retina relies on NAD-IDH and NADP-IDH (IDH2) cannot substitute for the defective NAD-IDH^13^. These human findings highlight the central role of NAD+ in PR degeneration via its intersecting position in redox homeostasis and metabolism^108,169^.

## Conclusions

Disruption of nutrient availability and metabolism has been suggested to be a unifying mechanism in PR death^13–17,170^. Many IRDs that are caused by a heterogenous group of genetic mutations can be recapitulated experimentally by mutating a number of key proteins involved in metabolism and redox homeostasis^13,56^. Acquired metabolic and oxidative dysfunctions have also been implicated in AMD^115,129^. Numerous studies on PR metabolism have been fruitful in determining the temporal mechanism of rod and cone PR degeneration, as well as inspiring innovative therapeutic strategies^44^. However, a more nuanced understanding of several areas of PR metabolism is likely to speed translational efforts and develop novel therapeutics to restore vision. These areas include the interconnection of metabolism among macromolecules, such as glucose, lipids, and nucleic acids, the union of metabolic and redox homeostasis, and the outer retinal microenvironment including nutrient sharing, competition, and signaling metabolites. In particular, forays into mitochondria-centric processes, along with amino acid metabolism, will deepen our understanding of the metabolic mechanisms underway and identify novel therapeutic targets for PR neuroprotection. Additionally, a more refined understanding of the interconnected nature between cellular metabolism and redox homeostasis is critical in the development of therapeutics aimed at protecting PRs. The importance of considering both metabolism and redox is illustrated by the unexpectedly high amount of aerobic glycolysis performed by PRs. From these various lenses, maintaining PR aerobic glycolysis may be the optimal strategy in limiting oxidative stress from OXPHOS and phototransduction, maximizing substrates available for anabolic biosynthesis, and providing the flexibility to shunt intermediates into different metabolic pathways to appropriately increase ATP production, redox response, or macromolecule production in the outer retinal metabolic microenvironment. Striking the most favorable balance between these competing interests may be the key to achieve successful neuroprotection in the retina. Thus, by deepening our understanding of metabolism, we may be able to reprogram these complex pathways to protect PRs from degeneration regardless of the disease.

### Reporting summary

Further information on research design is available in the Nature Research Reporting Summary linked to this article.

## Supplementary information

Reporting Summary

## References

[1] Wong WL (2014). Global prevalence of age-related macular degeneration and disease burden projection for 2020 and 2040: A systematic review and meta-analysis. Lancet Glob. Health.

[2] Li, J. Q. et al. Prevalence and incidence of age-related macular degeneration in Europe: a systematic review and meta-analysis. *Br. J. Ophthalmol*. 10.1136/bjophthalmol-2019-314422 (2019). 10.1136/bjophthalmol-2019-314422 31712255

[3] Bundey S, Crews SJ (1986). A study of retinitis pigmentosa in the city of Birmingham. J. Med. Genet..

[4] Farrar GJ (2017). Toward an elucidation of the molecular genetics of inherited retinal degenerations. Hum. Mol. Genet..

[5] Daiger, S. P., Rossiter, B. J. F., Greenberg, J., Christoffels, A. & Hide, W. *RetNet: Retinal Information Network*. https://sph.uth.edu/retnet/ (2020).

[6] Mitry D, Charteris DG, Fleck BW, Campbell H, Singh J (2010). The epidemiology of rhegmatogenous retinal detachment: geographical variation and clinical associations. Br. J. Ophthalmol..

[7] Rowe JA (1999). Retinal detachment in Olmsted County, Minnesota, 1976 through 1995. Ophthalmology.

[8] Haimann MH, Burton TC, Brown CK (1982). Epidemiology of retinal detachment. Arch. Ophthalmol..

[9] National Academies of Sciences, Engineering, and Medicine. *Making Eye Health a Population Health Imperative*. *Making Eye Health a Population Health Imperative* (National Academies Press, 2016). 27656731

[10] Russell S (2017). Efficacy and safety of voretigene neparvovec (AAV2-hRPE65v2) in patients with RPE65-mediated inherited retinal dystrophy: a randomised, controlled, open-label, phase 3 trial. Lancet.

[11] Holz FG (2018). Efficacy and safety of lampalizumab for geographic atrophy due to age-related macular degeneration: Chroma and spectri phase 3 randomized clinical trials. JAMA Ophthalmol..

[12] Hurley JB, Lindsay KJ, Du J (2015). Glucose, lactate, and shuttling of metabolites in vertebrate retinas. J. Neurosci. Res..

[13] Hartong DT (2008). Insights from retinitis pigmentosa into the roles of isocitrate dehydrogenases in the Krebs cycle. Nat. Genet..

[14] Bowne SJ (2006). Why do mutations in the ubiquitously expressed housekeeping gene IMPDH1 cause retina-specific photoreceptor degeneration?. Invest. Ophthalmol. Vis. Sci..

[15] Wang F (2014). A missense mutation in HK1 leads to autosomal dominant retinitis pigmentosa. Invest. Ophthalmol. Vis. Sci..

[16] Punzo C, Kornacker K, Cepko CL (2009). Stimulation of the insulin/mTOR pathway delays cone death in a mouse model of retinitis pigmentosa. Nat. Neurosci..

[17] Zhang L (2016). Reprogramming metabolism by targeting sirtuin 6 attenuates retinal degeneration. J. Clin. Invest..

[18] LaVail MM (1976). Rod outer segment disk shedding in rat retina: relationship to cyclic lighting. Science.

[19] Curcio CA (2018). Antecedents of soft drusen, the specific deposits of age-related macular degeneration, in the biology of human macula. Invest. Ophthalmol. Vis. Sci..

[20] Molday RS, Moritz OL (2015). Photoreceptors at a glance. J. Cell Sci..

[21] Fu Y, Yau K-W (2007). Phototransduction in mouse rods and cones. Pflug. Arch..

[22] Mustafi D, Engel AH, Palczewski K (2009). Structure of cone photoreceptors. Prog. Retin. Eye Res..

[23] Imamoto Y, Shichida Y (2014). Cone visual pigments. Biochim. Biophys. Acta Bioenerg..

[24] Snodderly DM, Sandstrom MM, Leung IYF, Zucker CL, Neuringer M (2002). Retinal pigment epithelial cell distribution in central retina of rhesus monkeys. Invest. Ophthalmol. Vis. Sci..

[25] Ames A, Li YY, Heher EC, Kimble CR (1992). Energy metabolism of rabbit retina as related to function: high cost of Na+ transport. J. Neurosci..

[26] Okawa H, Sampath AP, Laughlin SB, Fain GL (2008). ATP consumption by mammalian rod photoreceptors in darkness and in light. Curr. Biol..

[27] Ahmed J, Braun RD, Dunn R, Linsenmeier RA (1993). Oxygen distribution in the macaque retina. Invest. Ophthalmol. Vis. Sci..

[28] Landfried B (2017). Digoxin-induced retinal degeneration depends on rhodopsin. Cell Death Dis.

[29] Nikonov SS, Kholodenko R, Lem J, Pugh EN (2006). Physiological features of the S- and M-cone photoreceptors of wild-type mice from single-cell recordings. J. Gen. Physiol..

[30] Ingram NT, Fain GL, Sampath AP (2020). Elevated energy requirement of cone photoreceptors. Proc. Natl Acad. Sci. USA.

[31] Nihira M, Anderson K, Gorin FA, Burns MS (1995). Primate rod and cone photoreceptors may differ in glucose accessibility. Invest. Ophthalmol. Vis. Sci..

[32] Noell WK (1952). Electrophysiologic study of the retina during metabolic impairment. Am. J. Ophthalmol..

[33] Macaluso C, Onoe S, Niemeyer G (1992). Changes in glucose level affect rod function more than cone function in the isolated, perfused cat eye. Invest. Ophthalmol. Vis. Sci..

[34] Swarup A (2019). Modulating GLUT1 expression in retinal pigment epithelium decreases glucose levels in the retina: Impact on photoreceptors and müller glial cells. Am. J. Physiol. Cell Physiol..

[35] Kooragayala K (2015). Quantification of oxygen consumption in retina ex vivo demonstrates limited reserve capacity of photoreceptor mitochondria. Invest. Ophthalmol. Vis. Sci..

[36] Zhang L (2018). Whole-exome sequencing revealed HKDC1 as a candidate gene associated with autosomal-recessive retinitis pigmentosa. Hum. Mol. Genet..

[37] Pierrache LHM (2017). Whole-exome sequencing identifies biallelic IDH3A variants as a cause of retinitis pigmentosa accompanied by Pseudocoloboma. Ophthalmology.

[38] Fattal-Valevski A (2017). Homozygous mutation, p.Pro304His, in IDH3A, encoding isocitrate dehydrogenase subunit is associated with severe encephalopathy in infancy. Neurogenetics.

[39] Hartong DT, Berson EL, Dryja TP (2006). Retinitis pigmentosa. Lancet.

[40] Daiger SP, Bowne SJ, Sullivan LS (2007). Perspective on genes and mutations causing retinitis pigmentosa. Arch. Ophthalmol..

[41] Ullah I (2016). Mutations in phosphodiesterase 6 identified in familial cases of retinitis pigmentosa. Hum. Genome Var..

[42] Dryja TP (1995). Mutations in the gene encoding the α subunit of the rod cGMP-gated channel in autosomal recessive retinitis pigmentosa. Proc. Natl Acad. Sci. USA.

[43] Plana-Bonamaisó A (2020). Post-translational regulation of retinal IMPDH1 in vivo to adjust GTP synthesis to illumination conditions. elife.

[44] Aït-Ali N (2015). Rod-derived cone viability factor promotes cone survival by stimulating aerobic glycolysis. Cell.

[45] Venkatesh A (2015). Activated mTORC1 promotes long-term cone survival in retinitis pigmentosa mice. J. Clin. Invest..

[46] Du J (2013). Cytosolic reducing power preserves glutamate in retina. Proc. Natl Acad. Sci. USA.

[47] Petit L (2018). Aerobic glycolysis is essential for normal rod function and controls secondary cone death in retinitis pigmentosa. Cell Rep..

[48] Winkler BS, DeSantis N, Solomon F (1986). Multiple NADPH-producing pathways control glutathione (GSH) content in retina. Exp. Eye Res..

[49] Warburg O (1956). On the origin of cancer cells. Science.

[50] Vander Heiden MG, Cantley LC, Thompson CB (2009). Understanding the Warburg effect: the metabolic requirements of cell proliferation. Science.

[51] Wang L, Kondo M, Bill A (1997). Glucose metabolism in cat outer retina. Effects of light and hyperoxia. Invest. Ophthalmol. Vis. Sci..

[52] Winkler BS, Arnold MJ, Brassell MA, Sliter DFL (1997). Glucose dependence of glycolysis, hexose monophosphate shunt activity, energy status, and the polyol pathway in retinas isolated from normal (nondiabetic) rats. Invest. Ophthalmol. Vis. Sci..

[53] Winkler BS (1978). A role for metabolism in photoreceptor electrogenesis. Exp. Eye Res..

[54] Murray AR (2015). Glycosylation of rhodopsin is necessary for its stability and incorporation into photoreceptor outer segment discs. Hum. Mol. Genet..

[55] Murray AR, Fliesler SJ, Al-Ubaidi MR (2009). Rhodopsin: the functional significance of asn-linked glycosylation and other post-translational modifications. Ophthalmic Genet..

[56] Sinha T, Ikelle L, Naash MI, Al-Ubaidi MR (2020). The intersection of serine metabolism and cellular dysfunction in retinal degeneration. Cells.

[57] Scerri TS (2017). Genome-wide analyses identify common variants associated with macular telangiectasia type 2. Nat. Genet..

[58] Gantner ML (2019). Serine and lipid metabolism in macular disease and peripheral neuropathy. N. Engl. J. Med..

[59] Tuson M, Marfany G, Gonzàlez-Duarte R (2004). Mutation of CERKL, a novel human ceramide kinase gene, causes autosomal recessive retinitis pigmentosa (RP26).. Am. J. Hum. Genet..

[60] Wang W (2011). Selective rod degeneration and partial cone inactivation characterize an iodoacetic acid model of swine retinal degeneration. Invest. Ophthalmol. Vis. Sci..

[61] Kanow MA (2017). Biochemical adaptations of the retina and retinal pigment epithelium support a metabolic ecosystem in the vertebrate eye. elife.

[62] Lindsay KJ (2014). Pyruvate kinase and aspartate-glutamate carrier distributions reveal key metabolic links between neurons and glia in retina. Proc. Natl Acad. Sci. USA.

[63] Riepe R, Norenburg M (1977). Müller cell localisation of glutamine synthetase in rat retina. Nature.

[64] Wang W (2016). Two-step reactivation of dormant cones in retinitis pigmentosa. Cell Rep..

[65] Rueda EM (2016). The cellular and compartmental profile of mouse retinal glycolysis, tricarboxylic acid cycle, oxidative phosphorylation, and ~P transferring kinases. Mol. Vis..

[66] Swamy V, McGaughey D (2019). Eye in a disk: eyeintegration human pan-eye and body transcriptome database version 1.0. Invest. Ophthalmol. Vis. Sci..

[67] Bryan JM (2018). Identifying core biological processes distinguishing human eye tissues with precise systems-level gene expression analyses and weighted correlation networks. Hum. Mol. Genet..

[68] Kurihara T (2016). Hypoxia-induced metabolic stress in retinal pigment epithelial cells is sufficient to induce photoreceptor degeneration. elife.

[69] Zhao C (2011). mTOR-mediated dedifferentiation of the retinal pigment epithelium initiates photoreceptor degeneration in mice. J. Clin. Invest..

[70] Wang W (2019). Metabolic deregulation of the blood-outer retinal barrier in retinitis pigmentosa. Cell Rep..

[71] Philp NJ, Ochrietor JD, Rudoy C, Muramatsu T, Linser PJ (2003). Loss of MCT1, MCT3, and MCT4 expression in the retinal pigment epithelium and neural retina of the 5A11/basigin-null mouse. Invest. Ophthalmol. Vis. Sci..

[72] Philp NJ, Yoon H, Grollman E (1998). Monocarboxylate transporter MCT1 is located in the apical membrane and MCT3 in the basal membrane of rat RPE. Am. J. Physiol..

[73] Han JYS (2020). Role of monocarboxylate transporters in regulating metabolic homeostasis in the outer retina: Insight gained from cell-specific Bsg deletion. FASEB J..

[74] Daniele LL, Sauer B, Gallagher SM, Pugh EN, Philp NJ (2008). Altered visual function in monocarboxylate transporter 3 (Slc16a8) knockout mice. Am. J. Physiol. Cell Physiol..

[75] Fritsche LG (2013). Seven new loci associated with age-related macular degeneration. Nat. Genet..

[76] Ochrietor JD (2002). Inactivation of the Basigin gene impairs normal retinal development and maturation. Vis. Res..

[77] Wolf A (2011). Hexokinase 2 is a key mediator of aerobic glycolysis and promotes tumor growth in human glioblastoma multiforme. J. Exp. Med..

[78] Wubben TJ (2017). Photoreceptor metabolic reprogramming provides survival advantage in acute stress while causing chronic degeneration. Sci. Rep..

[79] Wubben TJ (2020). Small molecule activation of metabolic enzyme pyruvate kinase muscle isozyme 2, PKM2, circumvents photoreceptor apoptosis. Sci. Rep..

[80] Rajala A, Wang Y, Soni K, Rajala RVS (2018). Pyruvate kinase M2 isoform deletion in cone photoreceptors results in age-related cone degeneration. Cell Death Dis.

[81] Rajala A (2018). Pyruvate kinase M2 regulates photoreceptor structure, function, and viability. Cell Death Dis.

[82] Weh, E. et al. Hexokinase 2 is dispensable for photoreceptor development but is required for survival during aging and outer retinal stress. *Cell Death Dis*. 10.1038/s41419-020-2638-2 (2020). 10.1038/s41419-020-2638-2 PMC7272456 32499533

[83] Wilson JE (2003). Isozymes of mammalian hexokinase: structure, subcellular localization and metabolic function. J. Exp. Biol..

[84] Gershon TR (2013). Hexokinase-2-mediated aerobic glycolysis is integral to cerebellar neurogenesis and pathogenesis of medulloblastoma. Cancer Metab..

[85] Rajala A, Gupta VK, Anderson RE, Rajala RVS (2013). Light activation of the insulin receptor regulates mitochondrial hexokinase. A possible mechanism of retinal neuroprotection. Mitochondrion.

[86] Christofk HR (2008). The M2 splice isoform of pyruvate kinase is important for cancer metabolism and tumour growth. Nature.

[87] Rajala RVS, Rajala A, Kooker C, Wang Y, Anderson RE (2016). The warburg effect mediator pyruvate kinase M2 expression and regulation in the retina. Sci. Rep..

[88] Anastasiou D (2012). Pyruvate kinase M2 activators promote tetramer formation and suppress tumorigenesis. Nat. Chem. Biol..

[89] Gui DY, Lewis CA, Vander Heiden MG (2013). Allosteric regulation of PKM2 allows cellular adaptation to different physiological states. Sci. Signal..

[90] Chinchore Y, Begaj T, Wu D, Drokhlyansky E, Cepko CL (2017). Glycolytic reliance promotes anabolism in photoreceptors. elife.

[91] Zhang E (2020). PKM2 ablation enhanced retinal function and survival in a preclinical model of retinitis pigmentosa. Mamm. Genome.

[92] Casson RJ (2016). M-type pyruvate kinase isoforms and lactate dehydrogenase a in the mammalian retina: Metabolic implications. Invest. Ophthalmol. Vis. Sci..

[93] Laplante M, Sabatini DM (2012). mTOR signaling in growth control and disease. Cell.

[94] Jomary C, Cullen J, Jones SE (2006). Inactivation of the akt survival pathway during photoreceptor apoptosis in the retinal degeneration mouse. Invest. Ophthalmol. Vis. Sci..

[95] Ivanovic I (2011). Deletion of the p85α regulatory subunit of phosphoinositide 3-kinase in cone photoreceptor cells results in cone photoreceptor degeneration. Invest. Ophthalmol. Vis. Sci..

[96] Lin B, Xiong G, Yang W (2018). Ribosomal protein S6 kinase 1 promotes the survival of photoreceptors in retinitis pigmentosa. Cell Death Dis..

[97] McDougald DS, Papp TE, Zezulin AU, Zhou S, Bennett J (2019). AKT3 gene transfer promotes anabolic reprogramming and photoreceptor neuroprotection in a pre-clinical model of retinitis pigmentosa. Mol. Ther..

[98] Cheng, S.-Y. et al. Altered photoreceptor metabolism in mouse causes late stage age-related macular degeneration-like pathologies. *Proc. Natl. Acad. Sci. USA*10.1073/pnas.2000339117 (2020). 10.1073/pnas.2000339117 PMC7293639 32434914

[99] Lin SC, Hardie DG (2018). AMPK: sensing glucose as well as cellular energy status. Cell Metab..

[100] Xu L, Kong L, Wang J, Ash JD (2018). Stimulation of AMPK prevents degeneration of photoreceptors and the retinal pigment epithelium. Proc. Natl Acad. Sci. USA.

[101] Rabinovitch RC (2017). AMPK maintains cellular metabolic homeostasis through regulation of mitochondrial reactive oxygen species. Cell Rep..

[102] Byrne LC (2015). Viral-mediated RdCVF and RdCVFL expression protects cone and rod photoreceptors in retinal degeneration. J. Clin. Invest..

[103] Léveillard T (2004). Identification and characterization of rod-derived cone viability factor. Nat. Genet..

[104] Yang Y (2009). Functional cone rescue by RdCVF protein in a dominant model of retinitis pigmentosa. Mol. Ther..

[105] SparingVision. *SparingVision Receives European Orphan Designation for its Drug Candidate SPVN06 Dedicated to Inherited Retinal Dystrophies*. http://sparingvision.com/wp-content/uploads/2020/07/SparingVision_PR_European_Orphan_Designation_VDEF.pdf. (2020).

[106] Ban N (2013). Light-dark condition regulates sirtuin mRNA levels in the retina. Exp. Gerontol..

[107] Kubota S (2010). Resveratrol prevents light-induced retinal degeneration via suppressing activator protein-1 activation. Am. J. Pathol..

[108] Lin JB (2016). NAMPT-mediated NAD+ biosynthesis is essential for vision in mice. Cell Rep..

[109] Zhong L (2010). The histone deacetylase Sirt6 regulates glucose homeostasis via Hif1α. Cell.

[110] Nagao A, Kobayashi M, Koyasu S, Chow CCT, Harada H (2019). HIF-1-dependent reprogramming of glucose metabolic pathway of cancer cells and its therapeutic significance. Int. J. Mol. Sci..

[111] Hoang QV, Linsenmeier RA, Chung CK, Curcio CA (2002). Photoreceptor inner segments in monkey and human retina: Mitochondrial density, optics, and regional variation. Vis. Neurosci..

[112] Linsenmeier RA, Zhang HF (2017). Retinal oxygen: from animals to humans. Prog. Retin. Eye Res..

[113] Wang L, Tornquist P, Bill A (1997). Glucose metabolism in pig inner retina in darkness and light. Acta Physiol. Scand..

[114] Joyal JS (2016). Retinal lipid and glucose metabolism dictates angiogenesis through the lipid sensor Ffar1. Nat. Med..

[115] Joyal J-S, Gantner ML, Smith LEH (2018). Retinal energy demands control vascular supply of the retina in development and disease: The role of neuronal lipid and glucose metabolism. Prog. Retin. Eye Res..

[116] Cohen LH, Noell WK (1960). Glucose catabolism of rabbit retina before and after development of visual function. J. Neurochem..

[117] Grenell A (2019). Loss of MPC1 reprograms retinal metabolism to impair visual function. Proc. Natl Acad. Sci. USA.

[118] Bisbach CM (2020). Succinate can shuttle reducing power from the hypoxic retina to the O2-rich pigment epithelium. Cell Rep..

[119] Yam M (2019). Proline mediates metabolic communication between retinal pigment epithelial cells and the retina. J. Biol. Chem..

[120] He L, Poblenz AT, Medrano CJ, Fox DA (2000). Lead and calcium produce rod photoreceptor cell apoptosis by opening the mitochondrial permeability transition pore. J. Biol. Chem..

[121] Hutto RA (2020). Increasing Ca2+ in photoreceptor mitochondria alters metabolites, accelerates photoresponse recovery, and reveals adaptations to mitochondrial stress. Cell Death Differ..

[122] Finnemann SC, Bonilha VL, Marmorstein AD, Rodriguez-Boulan E (1997). Phagocytosis of rod outer segments by retinal pigment epithelial cells requires αvβ5 integrin for binding but not for internalization. Proc. Natl Acad. Sci. USA.

[123] Sun M (2006). Light-induced oxidation of photoreceptor outer segment phospholipids generates ligands for CD36-mediated phagocytosis by retinal pigment epithelium: A potential mechanism for modulating outer segment phagocytosis under oxidant stress conditions. J. Biol. Chem..

[124] Bazan NG (2007). Homeostatic regulation of photoreceptor cell integrity: significance of the potent mediator neuroprotectin D1 biosynthesized from docosahexaenoic acid: the proctor lecture. Invest Ophthalmol. Vis. Sci..

[125] Neale BM (2010). Genome-wide association study of advanced age-related macular degeneration identifies a role of the hepatic lipase gene (LIPC). Proc. Natl Acad. Sci. USA.

[126] Khan KN (2016). Differentiating drusen: drusen and drusen-like appearances associated with ageing, age-related macular degeneration, inherited eye disease and other pathological processes. Prog. Retin. Eye Res..

[127] Adijanto J (2014). The retinal pigment epithelium utilizes fatty acids for ketogenesis implications for metabolic coupling with the outer retina. J. Biol. Chem..

[128] Wanders RJ (2013). Peroxisomes in human health and disease: metabolic pathways, metabolite transport, interplay with other organelles and signal transduction. Subcell Biochem..

[129] Fu Z (2019). Dyslipidemia in retinal metabolic disorders. EMBO Mol. Med..

[130] Das Y, Baes M (2019). Peroxisomal disorders and retinal degeneration.. Adv. Exp. Med. Biol..

[131] Fletcher AL, Pennesi ME, Harding CO, Weleber RG, Gillingham MB (2012). Observations regarding retinopathy in mitochondrial trifunctional protein deficiencies. Mol. Genet. Metab..

[132] Li S (2013). Fatty acid transport protein 4 (FATP4) prevents light-induced degeneration of cone and rod photoreceptors by inhibiting RPE65 isomerase. J. Neurosci..

[133] Esteve-Rudd J (2018). Defective phagosome motility and degradation in cell nonautonomous RPE pathogenesis of a dominant macular degeneration. Proc. Natl Acad. Sci. USA.

[134] Gervois P, Torra IP, Fruchart J-C, Staels B (2000). Regulation of lipid and lipoprotein metabolism by PPAR activators Philippe. Clin. Chem. Lab. Med..

[135] Pearsall EA (2017). PPARα is essential for retinal lipid metabolism and neuronal survival. BMC Biol.

[136] Stahl A (2010). PPARγ mediates a direct antiangiogenic effect of ω3-PUFAs in proliferative retinopathy. Circ. Res..

[137] Chaube B (2015). AMPK maintains energy homeostasis and survival in cancer cells via regulating p38/PGC-1α-mediated mitochondrial biogenesis. Cell Death Discov..

[138] Alaynick WA (2008). Nuclear receptors, mitochondria and lipid metabolism. Mitochondrion.

[139] Zhang M (2018). Pgc-1α repression and high-fat diet induce age-related macular degeneration-like phenotypes in mice. DMM Dis. Model. Mech..

[140] Sarac O, Gulsuner S, Yildiz-Tasci Y, Ozcelik T, Kansu T (2012). Neuro-ophthalmologic findings in humans with quadrupedal locomotion. Ophthalmic Genet..

[141] Liu A, Chang J, Lin Y, Shen Z, Bernstein PS (2010). Long-chain and very long-chain polyunsaturated fatty acids in ocular aging and age-related macular degeneration. J. Lipid Res..

[142] Roomets E, Kivelä T, Tyni T (2008). Carnitine palmitoyltransferase i and Acyl-CoA dehydrogenase 9 in retina: insights of retinopathy in mitochondrial trifunctional protein defects. Invest. Ophthalmol. Vis. Sci..

[143] Harkewicz R (2012). Essential role of ELOVL4 protein in very long chain fatty acid synthesis and retinal function. J. Biol. Chem..

[144] Dai D-F, Chiao Y, Marcinek DJ, Szeto HH, Rabinovitch PS (2014). Mitochondrial oxidative stress in aging and healthspan. Longev. Health.

[145] Punzo C, Xiong W, Cepko CL (2012). Loss of daylight vision in retinal degeneration: are oxidative stress and metabolic dysregulation to blame?. J. Biol. Chem..

[146] Nandi A, Yan LJ, Jana CK, Das N (2019). Role of catalase in oxidative stress- and age-associated degenerative diseases. Oxid. Med. Cell. Longev.

[147] Ighodaro OM, Akinloye OA (2018). First line defence antioxidants-superoxide dismutase (SOD), catalase (CAT) and glutathione peroxidase (GPX): Their fundamental role in the entire antioxidant defence grid. Alex. J. Med..

[148] Adler L, Chen C, Koutalos Y (2014). Mitochondria contribute to NADPH generation in mouse rod photoreceptors. J. Biol. Chem..

[149] Kuehne A (2015). Acute activation of oxidative pentose phosphate pathway as first-line response to oxidative stress in human skin cells. Mol. Cell.

[150] Mullarky, E. & Cantley, L. C. Diverting Glycolysis to Combat Oxidative Stress. (eds Nakao, K., Minato, N. & Uemoto, S.) In *Innovative Medicine: Basic Research and Development [Internet]*. (Springer, Tokyo, 2015). 29787184

[151] Rotstein NP, Miranda GE, Abrahan CE, German OL (2010). Regulating survival and development in the retina: Key roles for simple sphingolipids. J. Lipid Res..

[152] Fujii T (2001). Immunohistochemical study of glutathione reductase in rat ocular tissues at different developmental stages. Histochem. J..

[153] Ursini F, Maiorino M (2020). Lipid peroxidation and ferroptosis: the role of GSH and GPx4. Free Radic. Biol. Med..

[154] Trachsel-Moncho L (2018). Oxidative stress and autophagy-related changes during retinal degeneration and development. Cell Death Dis..

[155] Ueta T (2012). Glutathione peroxidase 4 is required for maturation of photoreceptor cells. J. Biol. Chem..

[156] Lu L (2009). Increased expression of glutathione peroxidase 4 strongly protects retina from oxidative damage. Antioxid. Redox Signal.

[157] Xu R (2020). The retina and retinal pigment epithelium differ in nitrogen metabolism and are metabolically connected. J. Biol. Chem..

[158] Contreras, L. et al. Deficient glucose and glutamine metabolism in Aralar/AGC1/Slc25a12 knockout mice contributes to altered visual function. *Mol. Vis*. **22**, 1198–1212 (2016). PMC5063090 27746674

[159] Usui S (2011). Overexpression of SOD in retina: need for increase in H2O2-detoxifying enzyme in same cellular compartment. Free Radic. Biol. Med..

[160] Usui S (2009). Increased expression of catalase and superoxide dismutase 2 reduces cone cell death in retinitis pigmentosa. Mol. Ther..

[161] Lin JB, Apte RS (2018). NAD + and sirtuins in retinal degenerative diseases: a look at future therapies. Prog. Retin. Eye Res..

[162] Mills KF (2016). Long-term administration of nicotinamide mononucleotide mitigates age-associated physiological decline in mice. Cell Metab..

[163] Yoshida M (2019). Extracellular vesicle-contained eNAMPT delays aging and extends lifespan in mice. Cell Metab..

[164] Sasaki Y, Nakagawa T, Mao X, DiAntonio A, Milbrandt J (2016). NMNAT1 inhibits axon degeneration via blockade of SARM1-mediated NAD+ depletion. elife.

[165] Ozaki E (2020). SARM1 deficiency promotes rod and cone photoreceptor cell survival in a model of retinal degeneration. Life Sci. Alliance.

[166] Gerdts J, Brace EJ, Sasaki Y, DiAntonio A, Milbrandt J (2015). SARM1 activation triggers axon degeneration locally via NAD^+^ destruction. Science.

[167] Osterloh JM (2012). dSarm/Sarm1 is required for activation of an injury-induced axon death pathway. Science.

[168] Sasaki Y (2020). SARM1 depletion rescues NMNAT1-dependent photoreceptor cell death and retinal degeneration. elife.

[169] Imai, S. I. & Guarente, L. It takes two to tango: NAD+ and sirtuins in aging/longevity control. *npj Aging Mech. Dis*. **2**, 16017 (2016). 10.1038/npjamd.2016.17 PMC5514996 28721271

[170] Du, J., Linton, J. D. & Hurley, J. B. In *Methods in Enzymology* Vol. 561, (Elsevier Inc., 2015). 10.1016/bs.mie.2015.04.002 PMC6261279 26358904

[171] Wada Y (2005). Screening for mutations in the IMPDH1 gene in Japanese patients with autosomal dominant retinitis pigmentosa. Am. J. Ophthalmol..

[172] Kennan A (2002). Identification of an IMPDH1 mutation in autosomal dominant retinitis pigmentosa (RP10) revealed following comparative microarray analysis of transcripts derived from retinas of wild-type and Rho(−/−) mice. Hum. Mol. Genet..

[173] Samardzija M (2012). Activation of survival pathways in the degenerating retina of rd10 mice. Exp. Eye Res..

[174] Tuntivanich N (2009). Characterization of a canine model of autosomal recessive retinitis pigmentosa due to a PDE6A mutation. Invest. Ophthalmol. Vis. Sci..

[175] Sullivan LS (2014). A dominant mutation in hexokinase 1 (HK1) causes retinitis pigmentosa. Invest. Ophthalmol. Vis. Sci..

[176] Carrozzo R (2008). Peroxisomal acyl-CoA-oxidase deficiency: two new cases. Am. J. Med. Genet. A.

[177] Wang RY (2014). Effects of hematopoietic stem cell transplantation on acyl-CoA oxidase deficiency: a sibling comparison study. J. Inherit. Metab. Dis..

[178] Schrijver-Wieling I (1997). Retinal dystrophy in long chain 3-hydroxy-acyl-coA dehydrogenase deficiency. Br. J. Ophthalmol..

[179] Tyni T (1998). Ophthalmologic findings in long-chain 3-hydroxyacyl-CoA dehydrogenase deficiency caused by the G1528C mutation: a new type of hereditary metabolic chorioretinopathy. Ophthalmology.

[180] Zhang K (2001). A 5-bp deletion in ELOVL4 is associated with two related forms of autosomal dominant macular dystrophy. Nat. Genet..

[181] Barabas P (2013). Role of ELOVL4 and very long-chain polyunsaturated fatty acids in mouse models of Stargardt type 3 retinal degeneration. Proc. Natl Acad. Sci. USA.

[182] Findlay AS (2018). Mouse Idh3a mutations cause retinal degeneration and reduced mitochondrial function. Dis. Model. Mech.

[183] Koenekoop RK (2012). Mutations in NMNAT1 cause Leber congenital amaurosis and identify a new disease pathway for retinal degeneration. Nat. Genet..

[184] Falk MJ (2012). NMNAT1 mutations cause Leber congenital amaurosis. Nat. Genet..

[185] Greenwald SH (2016). Mouse models of NMNAT1-leber congenital amaurosis (LCA9) recapitulate key features of the human disease. Am. J. Pathol..

[186] Hoxhaj G, Manning BD (2020). The PI3K–AKT network at the interface of oncogenic signalling and cancer metabolism. Nat. Rev. Cancer.

